# Rectal and Vaginal Eradication of *Streptococcus agalactiae* (GBS) in Pregnant Women by Using *Lactobacillus salivarius* CECT 9145, A Target-specific Probiotic Strain

**DOI:** 10.3390/nu11040810

**Published:** 2019-04-10

**Authors:** Virginia Martín, Nivia Cárdenas, Sara Ocaña, María Marín, Rebeca Arroyo, David Beltrán, Carlos Badiola, Leónides Fernández, Juan M. Rodríguez

**Affiliations:** 1 Department of Nutrition and Food Science, Complutense University of Madrid, 28040 Madrid, Spain; vmartinmerino@gmail.com (V.M.); mlmarin@vet.ucm.es (M.M.); rebecaa@vet.ucm.es (R.A.); 2 Department of Galenic Pharmacy and Food Technology, Complutense University of Madrid, 28040 Madrid, Spain; niviacu@yahoo.com (N.C.); saripsima@yahoo.es (S.O.); leonides@vet.ucm.es (L.F.); 3 Unidad de Reproducción, Fundación Hospital Alcorcón, 28922 Alcorcón, Spain; 4 Centro de Diagnóstico Médico, Ayuntamiento de Madrid, 28006 Madrid, Spain; beltrangine@gmail.com; 5 Laboratorios Casen Recordati S.L., Vía de las Dos Castillas, 33, 28224 Pozuelo de Alarcón, Madrid, Spain; cjbadiola@casenrecordati.com

**Keywords:** Lactobacillus salivarius, Streptococcus agalactiae, GBS, probiotic, pregnancy

## Abstract

*Streptococcus agalactiae* (Group B Streptococci, GBS) can cause severe neonatal sepsis. The recto-vaginal GBS screening of pregnant women and intrapartum antibiotic prophylaxis (IAP) to positive ones is one of the main preventive options. However, such a strategy has some limitations and there is a need for alternative approaches. Initially, the vaginal microbiota of 30 non-pregnant and 24 pregnant women, including the assessment of GBS colonization, was studied. Among the *Lactobacillus* isolates, 10 *Lactobacillus salivarius* strains were selected for further characterization. In vitro characterization revealed that *L. salivarius* CECT 9145 was the best candidate for GBS eradication. Its efficacy to eradicate GBS from the intestinal and vaginal tracts of pregnant women was evaluated in a pilot trial involving 57 healthy pregnant women. All the volunteers in the probiotic group (*n* = 25) were GBS-positive and consumed ~9 log_10_ cfu of *L. salivarius* CECT 9145 daily from week 26 to week 38. At the end of the trial (week 38), 72% and 68% of the women in this group were GBS-negative in the rectal and vaginal samples, respectively. *L. salivarius* CECT 9145 seems to be an efficient method to reduce the number of GBS-positive women during pregnancy, decreasing the number of women receiving IAP during delivery.

## 1. Introduction

Neonatal sepsis contributes substantially to neonatal morbidity and mortality and is a major global public health challenge worldwide. According to the age of onset, neonatal sepsis is divided into early-onset sepsis (EOS) and late-onset sepsis (LOS). EOS has been variably defined based on the age at onset, with bacteremia or bacterial meningitis occurring at ≤72 h in infants hospitalized in the neonatal intensive care unit versus <7 days in term infants, and usually reflects transplacental or ascending infections from the maternal genitourinary tract [1].

*Streptococcus agalactiae* (Group B Streptococci, GBS) is one of the microorganisms most frequently involved in severe neonatal EOS cases [2,3,4]. Women, men and children of all ages can be asymptomatically colonized with GBS, acting the gastrointestinal tract, vagina and urethra as reservoirs. A recent systematic review and meta-analyses found that adjusted estimate for maternal GBS colonization worldwide was 18% (95% confidence interval [CI], 17%–19%), with regional variations (11%–35%) [5]. GBS vaginal and/or intestinal colonization is considered as a risk factor for ascending infection during pregnancy [6]. 

The relevance of GBS as an agent of neonatal infections soon prompted the finding of strategies for its eradication from the intestinal and genitourinary mucosal surfaces of pregnant women [7], including the use of chlorhexidine, which showed no effect [8] and, particularly, the development of vaccines. Unfortunately, no GBS vaccine is available at present despite the strong research efforts made in the last decades [9]. At present, two main approaches have been recommended for the prevention of neonatal GBS infections in Western countries: (a) a risk-based strategy; and (b) a screening-based strategy [10]. The second approach, involving recto-vaginal GBS screening at week 35–38 of pregnancy and subsequent intrapartum antibiotic prophylaxis (IAP) to positive mothers, is the preventive option followed in the USA and some European countries. 

However, such a strategy also faces some limitations: (a) it does not guarantee GBS eradication [11]; (b) it does not prevent GBS-related abortions, stillbirths and preterm births [4]; (c) it may lead to increasing rates of antibiotic resistance among GBS and other clinically relevant microorganisms [12,13,14]; and (d), it has a very negative impact on the acquisition, composition and development of the infant microbiota. Perinatal antibiotic use affects the gut microbiota development during the critical first weeks of life [15,16]. The composition of the gut microbiota of neonates whose mothers received IAP has been described as aberrant in comparison with that of non-treated neonates [17,18]. The detrimental impact of perinatal antibiotics, mainly IAP, on early life microbiota may have a lasting effect on the host’s health [19]. Therefore, there is a need for alternative strategies to avoid GBS colonization during pregnancy. 

In this context, the objective of this work was, first, the assessment of the presence of GBS in the vaginal exudate of healthy pregnant and non-pregnant women; and, second, the selection of a safe probiotic strain with the ability to eradicate GBS from the intestinal and genitourinary tracts of pregnant women.

## 2. Material and Methods

### 2.1. Microbiological Analysis of Vaginal Swabs Obtained from Pregnant and Non-pregnant Women

A total of 54 women (30 non-pregnant women and 24 pregnant women), aged 25–35, participated in this part of the study. In accordance with the Declaration of Helsinki, all volunteers gave written informed consent to the protocol, which had been approved (protocol 10/017-E) by the Ethical Committee of Clinical Research of the Hospital Clínico San Carlos Madrid (Spain). In relation to non-pregnant women, 4 vaginal exudates samples were collected within a menstrual cycle (days 0, 7, 14 and 21). Pregnant women provided a single sample in week 35–37 of pregnancy. All women claimed to be completely healthy. 

Samples were diluted in peptone water and spread onto Columbia Nalidixic Acid (CNA), Mac Conkey (MCK), Sabouraud Dextrose Chloramphenicol (SDC), Gardnerella (GAR) and Mycoplasma agar plates (BioMerieux, Marcy l’Etoile, France) for selective isolation and quantification of the main agents involved in vaginal infections. They were also spread onto agar plates of MRS (Oxoid, Basingstoke, UK) supplemented with either L-cysteine (2.5 g/L) (MRS-C) or horse blood (5%) (MRS-B) for isolation of lactobacilli. All the plates were incubated for 48 h at 37 °C in aerobic conditions, with the exception of the MRS-C and MRS-B ones, which were incubated anaerobically (85% nitrogen, 10% hydrogen, 5% carbon dioxide) in an anaerobic workstation (DW Scientific, Shipley, UK). Parallel, all the samples were submitted to an enrichment step in Todd Hewitt broth (Oxoid) to facilitate the isolation of *S. agalactiae* in CNA plates.

Initially, identification of the bacterial strains (at least one isolate of each colony morphology per medium and per sample) was performed by 16S rDNA sequencing using the primers and PCR conditions described by Kullen et al. [20]. Sequencing reactions were prepared using the ABI PRISM^®^ BigDye™ Terminator Cycle Sequencing kit with AmpliTaq DNA polymerase according to the manufacturer’s instructions (Applied Biosystems, Foster City, CA, USA) and were run on an ABI 377A automated sequencer (Applied Biosystems). The resulting sequences were used to search sequences deposited in the EMBL database using the BLAST algorithm. The identity of the strain was determined on the basis of the highest (>98%) scores. 

Identification of yeasts and confirmation of the initial 16S rDNA-based bacterial identifications was performed by MALDI-TOF (VITEK MS, BioMerieux, Marcy-L’Étoile, France) [21]. Identification of *S. agalactiae* isolates was also confirmed by using a latex agglutination test (Streptococcal grouping kit, Oxoid, Basingstoke, UK), following the instructions of the manufacturer.

Those isolates identified as belonging to the genus *Lactobacillus* were preserved for further studies. For such a purpose, an MRS-C broth culture of each isolate was mixed with glycerol (30%, v/v) and kept at −80 °C until required. A total of 89 different *Lactobacillus* strains were isolated from the vaginal swabs and submitted to the Random Amplification of Polymorphic DNA (RAPD) genotyping as described [22] in order to avoid duplication of isolates. Among them, 10 *Lactobacillus salivarius* strains were selected for further characterization on the basis of the following criteria: (1) absence of *S. agalactiae*, *Gardnella vaginalis, Candida spp., Ureaplasma* spp. and *Mycoplasma spp* in the vaginal samples from which the lactobacilli were originally isolated; (2) Qualified Presumption of Safety (QPS) status conceded by EFSA; and (3) the ability of the strain to grow rapidly in MRS broth under aerobic conditions (≥1 ×10^6^ cfu/mL after 16 h at 37 °C). 

### 2.2. Antimicrobial Activity of the Lactobacilli Strains against GBS

Initially, an overlay method [23] was used to determine the ability of the lactobacilli strains to inhibit the growth of 12 different *S. agalactiae* strains. Among them, 6 strains had been isolated from blood or cerebrospinal fluid in clinical cases of neonatal sepsis (Hospital Universitario Ramón y Cajal, Madrid, Spain) while the remaining 6 had been isolated from vaginal samples of pregnant women (our own collection). It was performed using MRS agar plates, on which the lactobacilli strains were inoculated as approximately 2 cm-long lines and incubated at 37 °C for 48 h. The plates were then overlaid with the indicator *S. agalactiae* strains vehiculated in 10 mL of Brain Heart Infusion (BHI, Oxoid) broth supplemented with soft agar (0.7%), at a concentration of ~10^4^ colony-forming units (cfu)/mL. The overlaid plates were incubated at 37 °C for 48 h and, then, examined for clear zones of inhibition (>2 mm) around the lactobacilli streaks. All experiments assaying inhibitory activity were performed in triplicate.

### 2.3. Production of Specific Antimicrobials (Bacteriocins, Lactic Acid, Hydrogen Peroxide) by the Lactobacilli Strains

Bacteriocin production was assayed using an agar diffusion method as described by Dodd et al. [24] and modified by Martín et al. [25], using the *S. agalactiae* strains as the indicator bacteria employed for the overlay method. The lactobacilli strains were screened for hydrogen peroxide production following the procedure described by Song et al. [26]. In the case of positive strains, hydrogen peroxide production was also measured by the quantitative method of Yap and Gilliland [27]. The concentration of L- and D-lactic acid in the supernatants of MRS cultures of the lactobacilli strains was quantified using an enzymatic kit (Roche Diagnostics, Mannheim, Germany), following the manufacturer’s instructions. The pH values of the supernatants were also measured. All these assays were performed in triplicate and the values were expressed as the mean ± SD. 

### 2.4. Coaggregation and Co-culture Assays

The ability of the lactobacilli strains to aggregate with cells of the *S. agalactiae* strains was investigated following the procedure of Younes et al. [28]. The suspensions were observed under a phase-contrast microscope after Gram staining.

To test the anti*-S. agalactiae* activity of the lactobacilli in a broth assay format, tubes containing 20 mL of MRS broth were co-inoculated with 1 mL of a *Lactobacillus* strain culture (7 log_10_cfu/mL) and 1 mL of an *S. agalactiae* strain (7 log_10_ cfu/mL). Subsequently, the cultures were incubated for 6 h at 37 °C in aerobic conditions. Immediately after the co-inoculation and after the incubation period, aliquots were collected, serially diluted and plated on MRS-C plates and CHROMagar StrepB agar plates (CHROMagar, París, France) for the selective enumeration of lactobacilli and streptococci, respectively. Correct taxonomic assignment was confirmed by the MALDI-TOF analysis as described previously. 

### 2.5. Survival After In Vitro Exposure to Saliva and Gastrointestinal-Like Conditions

The survival of the strain to conditions resembling those found in the human digestive tract (saliva, human stomach and small intestine) was assessed in the in vitro system described by Marteau et al. [29], with the modifications reported by Martín et al. [25]. For this purpose, the strain was vehiculated in UHT-treated milk (25 mL) at a concentration of 10^9^ CFU/mL. The values of the pH curve in the stomach-like compartment were those recommended by Conway et al. [30]. Different fractions were taken at 20, 40, 60, and 80 min from this compartment, and exposed for 120 minutes to a solution with a composition similar to that of human duodenal juice [30]. The survival rate of the strain was determined by culturing the samples on MRS agar plates, which were incubated at 37 °C for 48 h. 

### 2.6. Adhesion to Caco-2, HT-29 and Vaginal Cells and to Mucin

The ability of the strains to adhere to HT-29 and Caco-2 cells was evaluated as described by Coconnier et al. [31] with the modifications reported by Martín et al. [25]. HT-29 and Caco-2 were cultured to confluence in 2 mL of DMEM medium (PAA, Linz, Austria) containing 25 mM of glucose, 1 mM of sodium pyruvate and supplemented with 10% heat-inactivated fetal calf serum, 2 mM of L-glutamine and 1% non-essential amino acid preparation. At day 10 after confluence, 1 mL of the medium was replaced with 1 mL of DMEM containing 10^8^ CFU/mL of the strains. Adherence was measured as the number of lactobacilli adhered to the cells in 20 random microscopic fields. The assay was performed by triplicate.

Adherence to vaginal epithelial cells collected from healthy premenopausal women was performed as described previously [32]. 

The adhesion of the lactobacilli strains to mucin was determined according to the method described by Cohen and Laux [33].

### 2.7. Sensitivity to Antibiotics

The sensitivity of the strains to antibiotics was tested using the lactic acid bacteria susceptibility test medium (LSM) [34] and the microtiter VetMIC plates for lactic acid bacteria (National Veterinary Institute of Sweden, Uppsala, Sweden), as described previously [35]. Parallel, minimum inhibitory concentrations (MICs) were also determined by the E-test [AB BIODISK, Solna, Sweden) following the instructions of the manufacturer. Results were compared to the cut-off levels proposed by the European Food Safety Authority [36].

### 2.8. Hemolysis, Formation of Biogenic Amines and Degradation of Mucin

For investigation of hemolysis, strains were streaked onto layered fresh horse blood agar plates and grown for 24 h at 37 °C. Zones of clearing around colonies indicated hemolysin production. The capacity of the strains to synthesize biogenic amines (tyramine, histamine, putrescine and cadaverine) from their respective precursor amino acids (tyrosine, histidine, ornithine and lysine; Sigma-Aldrich) was evaluated using the method described by Bover-Cid and Holzapfel [37]. The potential of the strains to degrade gastric mucine (HGM; Sigma) was evaluated in vitro as indicated by Zhou et al. [38]. 

### 2.9. Acute and Repeated Dose (4-Weeks) Oral Toxicity Studies in a Rat Model

Wistar male and female rats (Charles River Inc., Marget, Kent, UK) were used to study the acute and repeated dose (4-weeks) oral toxicity of *L. salivarius* CECT 9145 in a rat model. Acclimation, housing and management (including feeding) of the rats was performed as previously described [39]. The rats were 56-days old at the initiation of treatment. Acute (limit test) and repeated dose (4 weeks) studies were conducted in accordance with the European Union guidelines (EC Council Regulation No. 440), and authorized by the Ethical Committee on Animal Research of the Complutense University of Madrid (protocol 240111). 

In the acute (limit test) study, 24 rats (12 males, 12 females) were distributed into two groups of 6 males and 6 females each. After an overnight of fasting, each rat received skim milk (500 µL) orally (control group or Group 1), or a single oral dose of 1 × 10^10^ CFU of *L. salivarius* CECT 9145 dissolved in 500 µL of skim milk (treated group or Group 2). Doses of the test and control products were administered by gavage. At the end of a 14 days observation period, the rats were weighed, euthanized by CO_2_ inhalation, exsanguinated, and necropsied.

The repeated dose (4 weeks) (limit test) study was conducted in 48 rats (24 males, 24 females) divided in four groups of 6 males and 6 females each (control group or Group 3; treated group or Group 4; satellite control group or Group 5; and satellite treated group or Group 6). Rats received a daily oral dose of either skim milk (Groups 3 and 5) or 1 × 10^9^ CFU of *L. salivarius* CECT 9145 dissolved in 500 µL of skim milk (Groups 4 and 6) for 4 weeks. All rats of Groups 3 and 4 were deprived of food for 18 h, weighed, euthanized by CO_2_ inhalation, exsanguinated, and necropsied on Day 29. All animals of the satellite groups (Groups 5 and 6) were kept a further 14 days without treatment to detect the delayed occurrence, persistence or recovery from potential toxic effects. All rats of the Groups 5 and 6 were deprived of food for 18 h, weighed, euthanized by CO_2_ inhalation, exsanguinated, and necropsied on day 42.

Behavior and clinical observations, blood biochemistry and hematology analysis, organ weight ratios and histopathological analysis were carried as described previously [39]. Bacterial translocation to blood, liver or spleen, and total liver glutathione (GSH) concentration was evaluated following the methods described by Lara-Villoslada et al. [40].

### 2.10. Efficacy of L. salivarius CECT 9145 to Eradicate GBS from the Intestinal and Vaginal Tracts of Pregnant Women: A Pilot Clinical Trial

In this prospective pilot clinical assay, 57 pregnant women (39 rectal and vaginal GBS-positive women; 18 rectal and vaginal GBS-negative women at the start of the intervention), aged 25–36, participated in this study. All met the following criteria: a normal pregnancy and a healthy status. Women ingesting probiotic supplements or receiving antibiotic treatment in the previous 30 days were excluded. Women with lactose intolerance or a cow’s milk protein allergy were also excluded because of the excipient used to administer the strain. All volunteers gave written informed consent to the protocol (10/017-E), which had been approved by the Ethical Committee of Clinical Research of the Hospital Clínico San Carlos Madrid (Spain). 

Volunteers were distributed into 3 groups (1 probiotic group and 2 placebo groups). All the volunteers in the probiotic group (*n* = 25) were GBS-positive and consumed a daily sachet with ~50 mg of freeze-dried probiotic (~9 log_10_ cfu of *L. salivarius* CECT 9145) from week 26 to week 38 of the pregnancy. Placebo subgroup 1 (*n* = 14) included GBS-positive women (pregnancy week ranging from 19 to 30) that were going to receive IAP because they had a previous baby that suffered a GBS sepsis. Placebo subgroup 2 (*n* = 18) included GBS-negative women (pregnancy week ranging from 14 to 26). Women in both placebo subgroups received a daily sachet containing 50 mg of the excipient used to carry the probiotic strain. In all cases, the intervention lasted from the start of the intervention to week 38. Probiotic- and excipient-containing sachets were kept at 4 °C throughout the study. All volunteers were provided with diaries to record compliance with the study product intake. Minimum compliance rate (% of the total treatment doses) was set at 86%.

Recto-vaginal GBS screening was performed at 28, 32 and 38 weeks. Rectal and vaginal exudates samples collected during the trial were serially diluted and plated on Granada (Biomerieux; isolation of hemolytic GBS, which appear as orange colonies), and CHROMagar StrepB (CHROMagar; for isolation of hemolytic and non- hemolytic GBS, which appear as purple colonies) agar plates. To avoid sensitivity-related problems, samples were submitted to a GBS enrichment step in Todd-Hewitt broth (Oxoid). After 24 h at 37 °C, the broth cultures were spread on CHROMagar agar plates. Correct taxonomic assignment was confirmed by MALDI-TOF and latex agglutination analyses, as described previously. At the last sampling time (week 38), recto-vaginal GBS screening was performed not only in our laboratory but also in those of the hospitals in which the respective women were going to deliver their babies.

Microbiological data were recorded as CFU/mL and transformed to logarithmic values before statistical analysis. Two-way ANOVA was used to investigate the effect of the individual (woman) and sampling time on the semiquantitative *S. agalactiae* counts in vaginal swabs. Statistical significance was set at *P* < 0.05. Statgraphics Centurion XVI version 17.0.16 (Statpoint Technologies Inc, Warrenton, Virginia) was used to carry out statistical analyses.

## 3. Results

### 3.1. Microbiological Analysis of Vaginal Swabs Obtained from Pregnant and Non-Pregnant Women

Bacterial growth was detected in all the samples when they were inoculated on MRS (2.70–8.08 log_10_ colony-forming units (cfu); mean 5.36 log_10_ cfu); CNA (3.00–7.92 log_10_ cfu; mean 5.13 log_10_ cfu) and GAR (2.70–8.10 log_10_ cfu; mean 5.24 log_10_ cfu) agar plates. Similar bacterial groups grew in these three media. Growth on MCK, SDC or Mycoplasma plates was only detected in a few percentages of samples (from 0% in Mycoplasma plates to ~40% in SDC plates).

*S. agalactiae* could be isolated from both non-pregnant (~25%) and pregnant (~19%) women. *Candida albicans* and other yeasts were isolated from approximately 7 and 36% of the non-pregnant and pregnant women, respectively. *Gardnerella vaginalis* was isolated in ~7% of the pregnant women. In both groups, *Lactobacillus* was the dominant genus since it was detected in ~93% of the participating women.

In relation to the samples provided by non-pregnant women, a total of 433 isolates (including at least one representative of each colony and cell morphology) were submitted to taxonomical analyses. The highest number of isolates corresponded to the genus *Lactobacillus* (28% of the total isolates), followed by *Staphylococcus* (17%)*, Enterococcus* (11%)*, Corynebacterium* (7%), and *Streptococcus* (4%). Among the *Lactobacillus* isolates, the main species were *L. gasseri* (24%), *L. crispatus* (23%), *L. salivarius* (21%), *L. vaginalis* (12%), *L. plantarum* (13%), *L. coleohominis* (5%), and *L. jensenii* (2%). Isolates belonging to the species *L. crispatus*, *L. gasseri*, *L. salivarius*, *L. vaginalis*, and *L. plantarum* could be isolated from the 4 phases of the menstrual cycle sampled in this study.

From the samples provided by pregnant women, 120 isolates were submitted to taxonomical analyses. Again, the genus *Lactobacillus* was associated to the highest number of isolates (17%), followed by *Staphylococcus* (15%)*, Streptococcus* (8%), yeasts (8%), *Enterococcus* (5%), *Bifidobacterium* (3%) and *Corynebacterium* (1%). Among the *Lactobacillus* isolates, the main species were *L. gasseri* (41%), *L. casei* (19%), *L. salivarius* (16%), *L. fermentum* (8%), *L. vaginalis* (6%), *L. reuteri* (5%) and *L. jensenii* (5%).

Among the *Lactobacillus* isolates obtained in this study, a few were selected to evaluate their potential as probiotics to control GBS populations on the basis of the following criteria: (1) absence of *S. agalactiae*, *Gardnella vaginalis, Candida* spp., *Ureaplasma* spp., and *Mycoplasma* spp. in the vaginal samples from which the lactobacilli were originally isolated; (2) Qualified Presumption of Safety (QPS) status (European Authority of Food Safety, EFSA); and (3) ability of the strain to grow rapidly in MRS broth under aerobic conditions (≥1 ×10^6^ cfu/mL after 16 h at 37 °C). In fact, only 10 strains (V3III-1, V4II-90, V7II-1, V7II-62, V7IV-1, V7IV 60, V8III-62, V11I-60, V11III-60 y, V11IV-60) met all the criteria and all of them belonged to the same species (*Lactobacillus salivarius*). These strains were then selected for further characterization. Later, *L. salivarius* V4II-90 was deposited in the Spanish Collection of Type Cultures (CECT) as *L. salivarius* CECT 9145 and, therefore, this is the name used for this strain in this article.

### 3.2. Antimicrobial Activity of the Lactobacilli Strains Against GBS and the Production of Potential Antimicrobial Compounds

Initially, the antimicrobial activity of the 10 selected lactobacilli against the *S. agalactiae* strains was determined by an overlay method. Clear inhibition zones (ranging from 2 to 20 mm) were observed around the lactobacilli streaks.

In relation to the antimicrobial compounds that may be responsible for such activity, the concentration of L- and D-lactic acid and the pH of the supernatants obtained from MRS cultures of the lactobacilli are shown in Table 1. The global concentration of L-lactic acid was similar (~10 mg/mL) in all the supernatants. In contrast, D-lactic acid was not detected in the supernatants of the tested strains. In addition, all the strains acidified the MRS-broth medium to a final pH of ~4 after 16 h of incubation; among them, *L. salivarius* V7IV-1 showed the highest acidifying capacity (final pH of 3.8). No bacteriocin-like activity could be detected against the tested *S. agalactiae* strains. Two strains (*L. salivarius* CECT 9145 and V7IV-1) were able to produce hydrogen peroxide (7.29 μg/mL ± 0.69 and 7.46 μg/mL ± 0.58, respectively) (Table 1).

**Figure 1:**
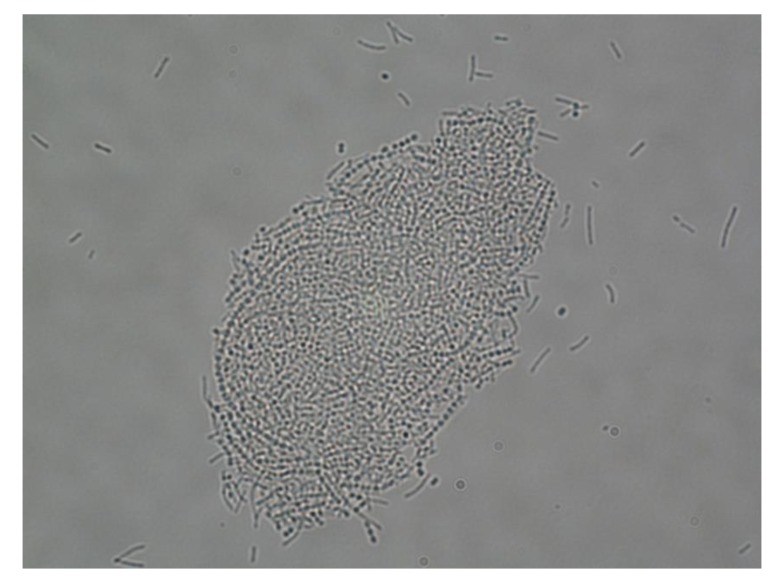
The strong co-aggregation between *L. salivarius* CECT 9145 (rods) and an *S. agalactiae* strain (cocci chains).

The capacity of the lactobacilli strains to form large well-defined co-aggregates with *S. agalactiae* was strain-dependent. Strains V3III-1, V7IV-60 and V11IV-60 coaggregated with 5 *S. agalactiae* strains; strains V8III-62, V11I-60 and V11III-60 with 7; strain V7II-62 with 9 *S. agalactiae* strains; and strains CECT 9145, V7II-1 and V7IV-1 with 10 *S. agalactiae* strains (Figure 1). The ability of the lactobacilli strains to interfere or inhibit the growth of four *S. agalactiae* strains was evaluated using MRS broth co-cultures. Co-cultures with *S. agalactiae* seemed not to affect the growth of any of the *L. salivarius* strains (Table 2). In contrast, most of the *L. salivarius* strains were able to interfere at a higher or lower degree with the growth of the different *S. agalactiae* strains included in this assay. Among them, *L. salivarius* CECT 9145 showed the highest ability to inhibit the growth of *S. agalactiae* since the presence of two of the four *S. agalactiae* strains was not detectable in the co-cultures and the concentration of the other two showed a ~2.5 log10 decrease after an incubation period of only 6 h at 37 ºC (Table 2). Interestingly, no viable streptococci could be detected when the co-cultures were incubated for 24 h (Table 2).

### 3.3. Survival After In Vitro Exposure to Saliva and Gastrointestinal-Like Conditions

The viability of the strains after exposition to conditions simulating those found in the gastrointestinal tract varied from ~64% (*L. reuteri* CR20, *L. salivarius* CECT 9145) to 30% (*L. salivarius* V3III-1) (Table 3).

### 3.4. Adhesion to Caco-2, HT-29 and Vaginal Cells and to Mucin

In this study, the lactobacilli strains tested were strongly adhesive to both Caco-2 and HT-29 cells, with the exception of the negative control strain (*L. casei imunitas*) which showed a low adhesive potential (Table 4). In addition, all showed adhesion to vaginal epithelial cells. Among the *L. salivarius* strains, *L. salivarius* CECT 9145 globally displayed the highest ability to adhere to both intestinal and vaginal epithelial cells (Table 4). The lactobacilli strains tested showed a variable ability to adhere to porcine mucin (Table 4). *L. salivarius* CECT 9145 and *L. salivarius* V7IV-1 were the strains that showed the highest adherence ability.

### 3.5. Sensitivity to Antibiotics

The MIC values of the lactobacilli strains for 16 antibiotics assayed are shown in Table 5. All the strains were sensitive to most of the antibiotics tested, including those considered clinically relevant antibiotics such as gentamycin, tetracycline, clindamycin, chloramphenicol, and ampicillin, showing MICs equal to or lower than the breakpoints defined by EFSA (EFSA, 2018). All the strains were resistant to vancomycin and kanamycin, which is an intrinsic property of the *L. salivarius* at the species level.

### 3.6. Hemolysis, the Formation of Biogenic Amines and the Degradation of Mucin

The strains did not show the ability to produce biogenic amines, and they were neither hemolytic nor able to degrade gastric mucin *in vitro*.

### 3.7. Acute and Repeated Dose (4 Weeks) Oral Toxicity Studies in a Rat Model

All animals survived both oral toxicity trials. The development of the treated animals during the experimental periods corresponded to their species and age. There were no significant differences in body weight or body weight gain among groups treated with *L. salivarius* CECT 9145 (including the satellite ones) in comparison to the control groups at any time point of the experimental period. No abnormal clinical signs, behavioral changes, body weight changes, hematological and clinical chemistry parameters, macroscopic or histological findings, or organ weight changes were observed. There were no statistical differences in body weights among groups. Similarly, no statistically significant differences in body weight gain, food and water consumption were observed between the groups. No significant differences in liver GSH concentration were observed between the control and treated groups (9.54 ± 1.21 vs. 9.37 ± 1.39 mmol/g, *P* > 0.1). *L. salivarius* CECT 9145 could be isolated from colonic material and vaginal swabs samples of all the treated animals (probiotic groups) at the end of the treatment. The concentration oscillated between 5.39 and 8.85 log_10_ cfu/g of the colonic material, and between 3.34 and 6.14 log_10_ cfu/swab in the vaginal samples. The strain could not be detected in any sample from the placebo group.

### 3.8. The Efficacy of L. salivarius CECT 9145 to Eradicate GBS from the Intestinal and Vaginal Tracts of Pregnant Women: A Pilot Clinical Trial

At the inclusion in the study, GBS was detected in both rectal and vaginal swabs obtained from 39 women, out of a total of 57 participating women, while the rest of the women (*n* = 18) were GBS-negative (Table 6). This last group of GBS-negative women, who did not ingest the *L. salivarius* strain also had negative GBS cultures from rectal and vaginal swabs taken regularly at 28, 32 and 36–38 weeks (Table 6). A group of GBS-positive women at the start of the study (*n* = 14) did not receive the probiotic and the routine screening results for vaginal and rectal GBS at 28, 32 and 36–38 weeks were found to be all positive (Table 6). 

Significantly, the group of GBS-positive women that started using the probiotic (9 log_10_ cfu/daily) since they were enrolled in this study (from 26 weeks) also tested positive for GBS at 28 weeks, but an increasing number of GBS-negative results appeared in the successive swabs collected until delivery (Table 6). At 30 weeks, the culture of rectal swabs taken from four women of this group rendered a negative result and the number of these samples increased to 18 (72% of the participants) at 38 weeks. Similar results were obtained by culturing vaginal swabs obtained from this group, although the proportion of women testing negative for GBS were always slightly higher when analyzing the rectal swabs than in vaginal swabs (Table 6).

**Figure 2:**
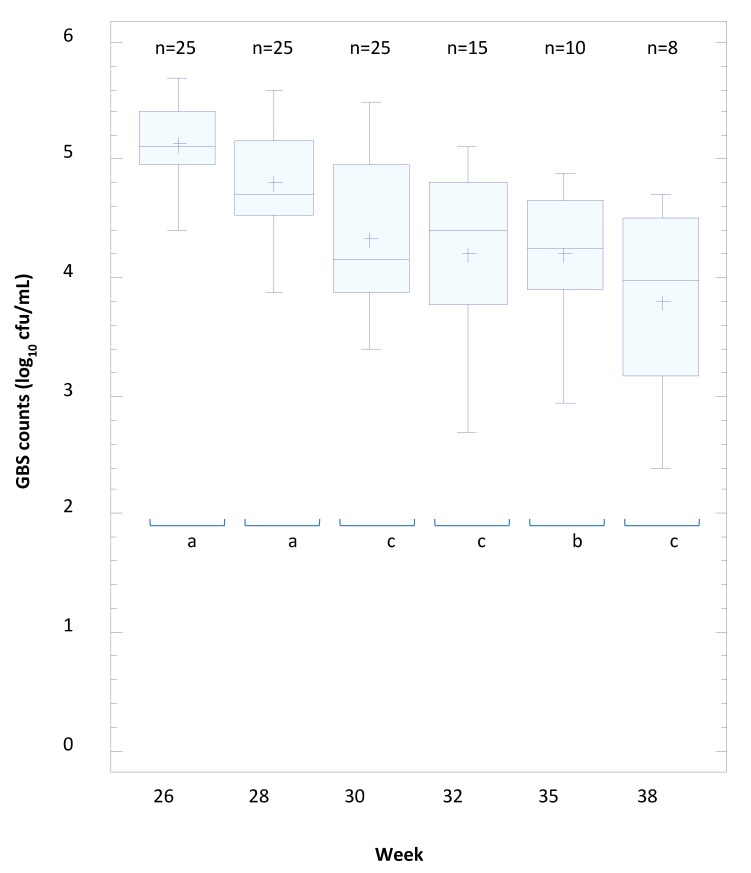
The mean concentrations (CFU/mL) of *S. agalactiae* (GBS) in vaginal swabs sampled regularly up to the delivery from Group B Streptococci (GBS)-positive women taking 9 log_10_ cfu of *L. salivarius* CECT 9145 daily. Statistically significant differences between samples taking at different sampling times are indicated by letters (Bonferroni post-hoc test).

The estimation of the concentration of GBS in vaginal swabs taken regularly up to the delivery from all participants is shown in Figure 2. There were no significant changes in both GBS-negative women (*n* = 18) and GBS-positive women (*n* = 14) without oral administration of *L. salivarius* CECT 9145 regarding the semiquantitative estimation of GBS. However, the number of vaginal swabs where GBS could not be detected increased in successive sampling times in the group that initially tested positive for GBS taking 9 log_10_cfu of *L. salivarius* CECT 9145 (*n* = 25). The mean value for *S. agalactiae* counts decreased significantly with the administration time *of L. salivarius* CECT 9145 (Figure 2) from a mean value of 5.14 cfu/mL at 26 weeks (*n* = 25) to 3.80 cfu/mL at 38 weeks (*n* = 9) (Figure 2).

No adverse effects arising from the intake of *L. salivarius* CECT 9145 were reported by any of the women who participated in this study. The results of the GBS status obtained in our laboratory at week 38 were identical to those obtained in the hospitals were the recruited women were screened for GBS and, as a result, none of the women who became GBS-negative in this study received IAP. 

## 4. Discussion

In this work, the GBS colonization rates were 25% and 20% among non-pregnant and pregnant women, respectively. In pregnant women, GBS colonization is found in up to 30% of rectovaginal samples [2,41] and stable colonization with the same clone for several years has been demonstrated [41]. Previous studies have shown that the presence of GBS is not linked to an abnormal microbiome or a reduction of the predominant *Lactobacillus* genus in the vaginal tract of the mother [42,43,44]. 

In contrast, a study involving a low number of participants found significant taxonomic differences in stools of 6-month infants, when mothers were GBS carriers, as compared to non-carriers [45]. Anyway, there is no epidemiological evidence for a correlation between neonatal colonization with GBS and specific shifts in the maternal intestinal or vaginal microbiome.

In the USA and many other countries (including Spain), women are routinely screened in the late third trimester (between 35 and 37 weeks’ gestation) for GBS colonization by rectovaginal swabs and subsequent cultures. If the rectovaginal swab is culture-positive, or if the patient has GBS in the urine, or has a prior history of GBS perinatal infection, intrapartum prophylactic antibiotics are administered to prevent vertical transmission of GBS to the neonate during labor and delivery. Some European countries (e.g., UK) have not adopted the GBS screening program but, instead, administer antibiotics upon the development of a risk factor for GBS neonatal disease (e.g., prolonged rupture of membranes). However, none of these approaches has eliminated neonatal GBS infections. This is because these prevention strategies do not address the risk of ascending infection, which can potentially occur anytime during pregnancy, leading to preterm birth or stillbirth. 

Overall, the prevention of GBS infection in pregnancy is still a complex question, with risk likely associated to several factors, including the pathogenicity of the GBS strain, host factors, influence of the vaginal/rectal microbiome, false-negative screening results, and/or changes in GBS antibiotic resistance [6,46]. Currently, strategies are mainly focused on the prevention of GBS transmission during labor and delivery through the use of antibiotics. This strategy does not fully capture the biology of the GBS infection, nor does it completely address the full burden of the GBS disease. Moreover, antibiotic resistance is increasing and the use of antibiotics during pregnancy has consequential effects for neonatal health that are only now being appreciated [47]. To successfully eradicate the burden of disease, interventions need to be specifically targeted while having minimal detrimental effects on the microbiome. Therefore, there is a need for alternatives that are respectful with the neonatal and infant microbiota, and that do not compromise the health of future generations. In this context, the final objective of this work was the selection of safe probiotic strains with the in vitro and in vivo ability to eradicate GBS from the intestinal and genitourinary tracts of pregnant women and/or their infants. 

The genus *Lactobacillus* constitutes the dominant bacterial group of the vaginal tract in most healthy women, playing a key role in the genitourinary homeostasis [48,49,50,51,52]. In this study, all the vaginal isolates (from either pregnant or non-pregnant healthy women) that fulfilled the initial selection criteria belonged to the species *L. salivarius.* This species is part of the indigenous microbiota of the human gastrointestinal tract, oral cavity, genitourinary tract and milk, and some strains have been studied as probiotics because of their in vitro and in vivo antimicrobial, anti-inflammatory and immunomodulatory properties [53,54,55,56,57,58,59,60,61,62,63,64]. Previous studies have shown the ability of *L. salivarius* strains to inhibit the growth of vaginal pathogens, including *Gardnerella vaginalis* and *Candida albicans* and, therefore, we have suggested their potential to be used as probiotics for the treatment or prevention of vaginal infections [65,66].

Administration of probiotic bacteria benefits the host through a wide array of mechanisms that are increasingly recognized as being either species- and/or strain-specific [67]. A comparative genomics study that included 33 *L. salivarius* strains isolated from humans, animals or food revealed that this species displays a high level of genomic diversity [68]. Therefore, the selection of *L. salivarius* strains for probiotic use requires the experimental validation of target-tailored phenotypic traits. Some *L. salivarius* strains have shown to be efficient in preventing infectious diseases such as mastitis caused by staphylococci and streptococci, when administered during late pregnancy [69]. Moreover, the oral administration of *L. salivarius* strains is also a valid strategy for the treatment of such a condition during lactation and, in fact, one of the strains was more efficient than antibiotics for this target [70]. In this work, the target was the antagonism towards GBS and, as a consequence, properties such as antimicrobial activity against *S. agalactiae* strains or coaggregation with this species were considered particularly relevant. 

The production of antagonistic substances such as bacteriocins, hydrogen peroxide or organic acids represents an important contribution to the defense mechanisms exerted by intestinal and vaginal lactobacilli [59,71]. Some *L. salivarius* strains produce bacteriocins or display bacteriocin-like activity against a variable spectrum of Gram-positive bacteria, including *S. agalactiae* strains [72]. However, none of the *L. salivarius* strains selected in our study displayed bacteriocin-like activity against *S. agalactiae* strains. Therefore, the antimicrobial activity that the selected *L. salivarius* strains exhibited against *S. agalactiae* must be related to the production of other antimicrobial compounds, such as organic acids. The ability of lactobacilli to acidify the vaginal milieu contributes to the displacement and inhibition of pathogens proliferation [73] and, more specifically, the acid production by lactobacilli has been directly correlated with the inhibition of GBS growth [74]. Another antimicrobial defense mechanism attributed to some intestinal or vaginal lactobacilli is the production of peroxide hydrogen, a compound that is toxic for catalase-negative bacteria, such as streptococci [75]. The production of this compound by *L. salivarius* has already been reported [59,76,77]. In our study, *L. salivarius* CECT 9145 (the strain that showed the highest anti-GBS activity) produced high amounts of lactic acid and, in addition, was able to produce peroxide hydrogen.

The ability to adhere to intestinal or vaginal epithelial cells or to mucin, and to co-aggregate with potential pathogens constitutes one of the main mechanisms for preventing their adhesion and colonization of mucosal surfaces. Therefore, it is not strange that such properties are considered relevant to the selection of probiotic strains [28]. The high adherence of *L. salivarius* strains to Caco-2 and HT-29 cells or to mucin has been previously observed [53,59,78]. Globally, *L. salivarius* CECT 9145 showed the best combination of adherence to epithelial cells, co-aggregation with *S. agalactiae* and the inhibition of *S. agalactiae* strains in broth co-cultures. This strain showed a high survival rate during transit through an in vitro gastrointestinal model and survival of lactobacilli when exposed to conditions found in the gastrointestinal tract seems to be a critical pre-requisite for a probiotic strain when its use as a food supplement is pursued, as it was the case.

Some vaginal strains of *L. gasseri* and *L reuteri* have also been reported to co-aggregate with GBS [78]. In contrast, no co-aggregation activity between *S. agalactiae* and other vaginal lactobacilli belonging to the species *L. acidophilus*, *L. gasseri* and *L. jensenii* was observed in another study [32], a fact suggesting that such property is a highly strain-specific trait. In relation to broth co-cultures, the capacity to antagonize the growth of *S. agalactiae* by lactobacilli strains belonging to different species, including *L. salivarius*, has been previously reported [79,80]. Similar to our results, this activity was strain-dependent [79]. 

One of the most important criteria for the selection of probiotic strains is the assessment of their safety, particularly to the target population. In this work, no adverse effect was reported by any of the women who participated in the clinical trial and ascribed to the probiotic group [thus, receiving *L. salivarius* CECT 9145 at 9 log_10_ cfu daily for several weeks). Previously, other *L. salivarius* strains have been shown to be well-tolerated and safe in animal models [40] and in human clinical assays [70,81,82,83], including one involving pregnant women [69].

The *L. salivarius* strains included in this study were very susceptible to most of the antimicrobials tested. In fact, their MICs were lower than the cut-offs established for lactobacilli to seven out of the eight antibiotics required for this species by the European Food Safety Authority [36]. The only exception was kanamycin. The intrinsic resistance of lactobacilli to kanamycin and other aminoglycosides (such as neomycin or streptomycin) has been repeatedly reported [84,85], and this is thought to be an *L. salivarius* species-specific trait due to the lack of cytochrome-mediated transport of this class of antibiotics [86]. The *L. salivarius* strains were also resistant to vancomycin but the assessment of vancomycin sensitivity is not required by EFSA in the case of homofermentative lactobacilli (including *L. salivarius*) since they are intrinsically resistant to this antibiotic probably due to the presence of D-Ala-D-lactate in their peptidoglycan structure [87]. Therefore, *L. salivarius* CECT 9145 and the other strains evaluated in this study can be considered as safe from this point of view.

Lactobacilli are among the Gram-positive bacteria with the potential to produce biogenic amines and these substances can cause several toxicological problems and/or may act as potential precursors of carcinogenic nitrosamines [37]. The screened *L. salivarius* strains neither produced histamine, tyramine, putrescine or cadaverine nor harbored the gene determinants required for their biosynthesis. Additionally, they were unable to degrade gastric mucin in vitro. 

Some studies have been focused on the potential of different lactic acid bacteria strains or their metabolites to inhibit the growth of *S. agalactiae* in vitro or in murine models [74,80,88,89,90,91,92,93,94,95]. However, few studies have evaluated the efficacy of probiotic strains for the rectal and vaginal eradication of GBS in pregnant women. Ho et al. [96] examined the effect of *Lactobacillus rhamnosus* GR-1 and *Lactobacillus reuteri* RC-14 taken orally on GBS-positive pregnant women at 35–37 weeks of gestation, and found that GBS colonization changed from positive to negative in 42.9% of the women in the probiotic group. The rate of women that became GBS-negative was lower than in our study and this might be due to the fact that, in the cited study, the recruited women started the probiotic intake many weeks later. A second study using the same two strains (*L. rhamnosus* GR-1 and *L. reuteri* RC-14) provided non-conclusive results due to the low adherence to the probiotic treatment since only seven of 21 women in the intervention group completed the entire 21 days of probiotics [97].

It is important to highlight that nutrition may also play a key role in creating mucosal conditions favoring the action of bacterial strains that are able to improve the rectal and vaginal environments, as it is the case of *L. salivarius* CECT 9145. Such conditions may include the selective fermentation of dietary fiber, the production of relevant bioactive compounds, such as short-chain fatty acids [98], or the use of hyaluronic acid, which has been shown to be useful in the treatment of female recurrent genitourinary infections [99]. The impact of diet on the outcomes of clinical assays involving probiotic-interventions is often underrated and should be taken into account in future studies.

Our study includes the whole process from strain isolation to a pilot clinical study specifically targeting GBS eradication in pregnant women. The criteria followed for the selection of the best candidate for such a target (*L. salivarius* CECT 9145) allowed a notable reduction in the rate of GBS-colonized women and led to a reduction in the use of antibiotics during the peripartum period. As a conclusion, the administration of *L. salivarius* CECT 9145 to GBS-positive pregnant women is a safe and successful strategy to significantly decrease the rates of GBS colonization during pregnancy and, therefore, to reduce the exposure of pregnant women and their infants to intrapartum prophylaxis. Work is in progress to study the mechanisms involving GBS antagonism, including the study of the strain genome and to initiate a multicenter well-designed clinical trial involving a higher number of women.

## Figures and Tables

**Table 1:** The pH and concentrations of L- and D-lactic acid (mg/mL; mean ± SD), and hydrogen peroxide (μg/mL; mean ± SD) in the supernatants obtained from the MRS cultures of the lactobacilli (*n* = 4).

Strain	pH	L –lactic Acid	D-lactic Acid	Hydrogen Peroxide
*L. salivarius* V3III-1	4.00	9.66 ± 0.57	Nd	Nd
*L. salivarius* CECT 9145	4.01	10.03 ± 0.60	Nd	7.29 ± 0.69
*L. salivarius* V7II-1	4.02	9.82 ± 0.69	Nd	Nd
*L. salivarius* V7II-62	4.01	9.76 ± 0.54	Nd	Nd
*L. salivarius* V7IV-1	3.85	10.47 ± 0.58	Nd	7.46 ± 0.58
*L. salivarius* V7IV-60	4.02	9.72 ± 0.63	Nd	Nd
*L. salivarius* V8III-62	4.04	9.91 ± 0.55	Nd	Nd
*L. salivarius* V11I-60	4.03	9.84 ± 0.43	Nd	Nd
*L. salivarius* V11III-60	4.07	9.61 ± 0.47	Nd	Nd
*L. salivarius* V11IV-60	4.03	10.02 ± 0.62	Nd	Nd
*L. salivarius* CECT 5713	3.93	10.26 ± 0.62	Nd	-

The initial pH value of MRS broth was 6.2. Nd: not detectable.

**Table 2:** The bacterial counts (log_10_ cfu/mL) of the *S. agalactiae* strains when co-cultured with the *L. salivarius* strains in MRS broth for 0, 6 and 24 h at 37 °C.

*L. salivarius* (Strain)	*S. agalactiae* (Strain)	0 h	6 h	24 h
V3III-1	RC5	7.10	6.44	Nd
	RC6	7.24	7.04	Nd
	V2I-80	7.10	7.04	Nd
	V14I-63	7.27	7.10	Nd
CECT 9145	RC5	7.04	4.48	Nd
	RC6	7.23	Nd	Nd
	V2I-80	7.10	4.70	Nd
	V14I-63	7.34	Nd	Nd
V7II-1	RC5	7.15	7.27	Nd
	RC6	7.15	6.70	Nd
	V2I-80	7.04	7.10	Nd
	V14I-63	7.35	5.65	Nd
V7II-62	RC5	7.24	7.04	Nd
	RC6	6.98	7.49	Nd
	V2I-80	7.35	7.92	Nd
	V14I-63	7.10	6.93	Nd
V7IV-1	RC5	7.32	7.58	Nd
	RC6	7.34	6.90	Nd
	V2I-80	7.15	7.38	Nd
	V14I-63	7.23	6.04	Nd
V7IV-60	RC5	7.24	7.32	Nd
	RC6	7.32	8.06	Nd
	V2I-80	7.04	7.15	Nd
	V14I-63	7.35	8.34	Nd
V8III-62	RC5	7.15	7.90	Nd
	RC6	7.34	7.23	Nd
	V2I-80	7.24	6.90	Nd
	V14I-63	7.20	8.77	Nd
V11I-60	RC5	7.31	7.44	Nd
	RC6	7.01	6.94	Nd
	V2I-80	7.23	7.07	Nd
	V14I-63	6.93	6.60	Nd
V11 III-60	RC5	7.27	6.44	Nd
	RC6	6.95	6.88	Nd
	V2I-80	7.28	6.52	Nd
	V14I-63	7.37	6.85	Nd
	RC5	7.26	6.74	Nd
V11IV-60	RC6	7.42	6.60	Nd
	V2I-80	7.10	6.60	Nd
	V14I-63	7.06	5.32	Nd
	RC5	7.20	9.32	9.34
Control cultures (no *L. salivarius* strain)	RC6	7.31	9.20	9.27
	V2I-80	7.04	9.15	9.23
	V14I-63	7.10	9.02	9.15

Nd: *S. agalactiae* was not detected.

**Table 3:** The percentage (%) of initial lactobacilli (9 log_10_ cfu/mL) that survived to conditions simulating those of the gastrointestinal tract.

Strain	% Total *
*L. salivarius* V3III-1	30.2 ^a^
*L. salivarius* CECT 9145	64.3 ^b^
*L. salivarius* V7II-1	59.8 ^b^
*L. salivarius* V7II-62	50.5 ^b^
*L. salivarius* V7IV-1	48.1 ^b^
*L. salivarius* V7IV-60	53.3 ^b^
*L. salivarius* V8III-62	41.3 ^c^
*L. salivarius* V11I-60	40.8 ^c^
*L. salivarius* V11III-60	41.1 ^c^
*L. salivarius* V11IV-60	42.3 ^c^
*L. salivarius* CELA2	64.4 ^b^

*, different letters mean statistically different values.

**Table 4:** The ability of the lactobacilli to adhere to HT-29, Caco-2 and vaginal epithelial cells, and to porcine mucin.

Strain	HT-29 ^a^	Caco-2 ^a^	Vaginal Cells ^b^	Adhesion ^c^
*L. salivarius* V3III-1	877.3 ± 303.2	259.1 ± 67.1	+	9.3 ± 2.0
*L. salivarius* CECT 9145	905.2 ± 297.0	345.1 ± 72.8	+++	10.9 ± 1.8
*L. salivarius* V7II-1	900.5 ± 336.2	297.8 ± 84.5	++	8.9 ± 1.9
*L. salivarius* V7II-62	911.7 ± 250.9	321.5 ± 80.2	++	9.0 ± 1.6
*L. salivarius* V7IV-1	884.0 ± 226.3	252.3 ± 67.1	++	8.5 ± 1.2
*L. salivarius* V7IV-60	799.7 ± 210.1	255.9 ± 60.3	++	9.6 ± 1.7
*L. salivarius* V8III-62	623.4 ± 200.2	108.7 ± 24.3	+	3.3 ± 0.7
*L. salivarius* V11I-60	593.2 ± 191.5	121.6 ± 22.0	+	2.9 ± 0.8
*L. salivarius* V11III-60	612.4 ± 188.2	153.2 ± 26.7	+	2.4 ± 1.0
*L. salivarius* V11IV-60	601.6 ± 172.0	159.5 ± 23.4	+	3.4 ± 0.8

^a^ The adherent lactobacilli in 20 random microscopic fields were counted for each test (*n* = 4). ^b^ Semiquantitative scale: 0, no adhesion; +, low adhesion; ++, middle adhesion; +++, high adhesion. ^c^ Values are expressed as the percentage of the fluorescence retained in the wells after the washing steps of the assay.

**Table 5:** The minimal inhibitory concentration (MIC, mg/mL) values of 16 antibiotics ^a^ to the *L. salivarius* strains.

	Antibiotic ^a^															
**Strain**	GEN	KAN	STP	NEO	TET	ERY	CLI	CHL	AMP	PEN	VAN	VIR	LIN	TRM	CIP	RIF
V3III-1	4	64	32	8	2	0.12	0.5	2	0.5	0.12	>128	0.5	0.5	0.5	2	0.5
CECT 9145	4	256	32	8	2	0.12	0.5	2	0.5	0.12	>128	0.5	1	0.25	4	1
V7II-1	4	128	32	4	2	0.12	0.5	4	0.5	0.12	>128	0.5	0.5	0.5	2	0.25
V7II-62	2	128	32	8	2	0.25	0.5	2	0.5	0.25	>128	0.25	1	0.25	2	0.5
V7IV-1	8	256	32	4	2	0.12	0.5	2	0.5	0.25	>128	0.5	1	0.5	2	0.5
V7IV-60	8	128	32	8	2	0.12	0.4	4	0.5	0.25	>128	0.5	1	0.5	2	0.5
V8III-62	8	128	32	2	2	0.25	0.5	4	0.5	0.25	>128	1	1	0.5	2	0.5
V11I-60	4	128	32	8	2	0.12	0.5	2	0.5	0.25	>128	1	1	0.5	2	0.5
V11III-60	8	256	32	4	2	0.12	0.5	2	0.5	0.25	>128	0.5	1	0.5	2	0.5
V11IV-60	4	128	32	8	2	0.12	0.5	2	0.5	0.25	>128	1	1	0.5	2	0.5
Breakpoint ^b^	16	64 (R)	64	nr	8	1	4	4	4	nr	nr (R)	nr	nr	nr	nr	nr

^a^ Abbreviations: GEN, gentamycin; KAN, kanamycin; STP, streptomycin; NEO, neomycin; TET, tetracycline; ERY, erythromycin; CLI, clindamycin; CHL, chloramphenicol; AMP, ampicillin; PEN, penicillin; VAN, vancomycin; VIR, virginiamycin; LIN, linezolid; TRM, trimethoprim; CIP, ciprofloxacin; RIF, rifampicin; nr, not required by EFSA. R, the species *L. salivarius* is intrinsically resistant. ^b^ Breakpoint: microbiological breakpoints (mg/mL) that categorise *Lactobacillus salivarius* as resistant (microbiological breakpoints are defined as the MIC values that clearly deviate from those displayed by the normal susceptible populations; EFSA, 2018).

**Table 6:** The qualitative assessment (Group B Streptococci (GBS)-positive/GBS-negative) of *Streptococcus agalactiae* in rectal and vaginal swabs of participants (*N* = 57).

Initial GBS Status	Probiotic Intake	GBS Status	Rectal Swabs (Week)						Vaginal Swabs (Week)					
Negative	NO		12-26	28 ^a^		32 ^b^		36-38	12-26	28 ^a^		32 ^b^		36–38
(*n* = 18)		GBS-positive	0	0		0		0	0	0		0		0
		GBS-negative	18	17		16		18	18	17		16		18
		GBS-negative (%)	100	100		100		100	100	100		100		100
Positive	NO		14–17	28 ^b^		32 ^a^		36–38	14–17	28 ^b^		32 ^a^		36–38
(*n* = 14)		GBS-positive	14	12		13		14	14	12		13		14
		GBS-negative	0	0		0		0	0	0		0		0
		GBS-negative (%)	0	0		0		0	0	0		0		0
Positive	YES		26	28	30	32	35	38	26	28	30	32	35	38
(*n* = 25)		GBS-positive	25	25	21	12	9	7	25	25	25	15	10	8
		GBS-negative	0	0	4	13	16	18	0	0	0	10	15	17
		GBS-negative (%)	0	0	16	52	64	72	0	0	0	40	60	68

^a^: sample from one participant was missing at this sampling time. ^b^: samples from two participants were missing at this sampling time.
